# Multiple Mononeuropathy Secondary to Parvovirus B19 Infection: A Case Series

**DOI:** 10.1111/ene.70565

**Published:** 2026-03-20

**Authors:** Julian Theuriet, Maud Michaud, Guillaume Fargeot, Céline Labeyrie, Anaïs Grosset, Maude Bucy, Ludivine Kouton, Florian Hubben, Véronique Manel, Florent Cluse, Adrien Bohic, Nicolas Rodriguez, Philippe Petiot, Geneviève Billaud, Vincent Fabry, Pascal Cintas, Thierry Maisonobe, Karine Viala, Rabab Debs, Dimitri Psimaras, Sarah Leonard‐Louis, Benjamin Terrier, Alina Dorobat, Céline Tard, Stéphane Darteyre, Alex Vicino, Marie Théaudin, Clovis Adam, Françoise Bouhour, Timothée Lenglet, Grégory Destras, Nathalie Streichenberger, Antoine Pegat

**Affiliations:** ^1^ Service d'ENMG et de Pathologies Neuromusculaires, Centre de Référence des Maladies Neuromusculaires PACA‐Réunion‐Rhône‐Alpes Hôpital Neurologique Pierre Wertheimer, Hospices Civils de Lyon, Groupement Est Bron France; ^2^ Pathophysiology and Genetics of Neuron and Muscle, CNRS UMR 5261, INSERM U1315 Université Claude Bernard Lyon 1, Faculté de Médecine Lyon Est Lyon France; ^3^ Service de Neurologie, CRMR Maladies Neuromusculaires Rares NEIDF Hôpital Central, CHRU de Nancy Nancy France; ^4^ Service de Neurologie, Hôpital Bicêtre Assistance Publique Hôpitaux de Paris Le Kremlin Bicêtre France; ^5^ Service de Pathologies Neuromusculaires Hôpital de la Timone, Assistance Publique des Hôpitaux de Marseille Marseille France; ^6^ Service de Neurologie – Sclérose en Plaques, Pathologies de la Myéline et Neuro‐Inflammation Hôpital Neurologique Pierre Wertheimer, Hospices Civils de Lyon, Groupement Est Bron France; ^7^ Service de Médecine Physique et Réadaptation Pédiatrique, L'Escale Hôpital Femme‐Mère‐Enfant, Hospices Civils de Lyon, Groupement Est Bron France; ^8^ Service de Neurologie, Troubles du Mouvement et Pathologies Neuromusculaires Hôpital Neurologique Pierre‐Wertheimer, Hospices Civils de Lyon, Groupement Est Bron France; ^9^ Laboratoire de Virologie Hôpital de la Croix‐Rousse, Hospices Civils de Lyon, Groupement Nord Lyon France; ^10^ Service de Neurologie, CHU de Toulouse Toulouse France; ^11^ Département de Neurophysiologie Clinique Hôpital Pitié‐Salpêtrière, Assistance Publique – Hôpitaux de Paris Paris France; ^12^ Département de Neuropathologie Hôpital Pitié‐Salpêtrière, Assistance Publique – Hôpitaux de Paris Paris France; ^13^ Service de Neuro‐Oncologie Hôpital Pitié‐Salpêtrière, Assistance Publique – Hôpitaux de Paris Paris France; ^14^ Centre de Référence des Maladies Neuromusculaires Nord/Est/Ile de France, Institut de Myologie Hôpital Pitié‐Salpêtrière, Assistance Publique des Hôpitaux de Paris Paris France; ^15^ Service de Médecine Interne Hôpital Cochin, Assistance Publique – Hôpitaux de Paris Paris France; ^16^ Service de Neurologie, CH Montluçon Montluçon France; ^17^ Service de Neurologie, U1172 Centre de Référence des Maladies Neuromusculaires Nord/Est/Ile de France, CHU de Lille Lille France; ^18^ Service de Neurologie Pédiatrique, Département Femme‐Mère‐Enfant CHU Lausanne Lausanne Switzerland; ^19^ Nerve Muscle Unit, Service of Neurology, Department of Clinical Neurosciences University Hospital of Lausanne and Lausanne University Hospital Lausanne Switzerland; ^20^ Département de Neuropathologie Hôpital Bicêtre, Assistance Publique – Hôpitaux de Paris Le Kremlin Bicêtre France; ^21^ Service de Neuropathologie Hospices Civils de Lyon, Groupement Est Bron France

**Keywords:** mononeuropathy multiplex, multiple mononeuropathy, neuropathy, parvovirus B19, vasculitis

## Abstract

**Background:**

Parvovirus B19 (B19V) infection has been associated with neurological complications. Rarely, patients present with multiple mononeuropathy (MM). The present study aimed to better characterize the clinical, electrophysiological, and prognostic features of patients with B19V‐related MM.

**Methods:**

This retrospective, observational, multicenter study included patients with B19V‐related MM diagnosed between January 2015 and January 2025 in seven university hospitals in France and Switzerland.

**Results:**

Twenty‐one patients were included. Twelve were female (57%). All were immunocompetent. The median age at symptom onset was 40 years [IQR: 31–44]. All patients experienced sensory symptoms, 19 (90%) reported neuropathic pain, nine (43%) developed motor weakness, and seven (33%) had cranial nerve involvement. The most frequently involved nerves were the median (14 patients, 67%), fibular (*n* = 13; 62%), and ulnar (*n* = 10; 48%) nerves. B19V IgM antibodies were present in 12/20 patients (60%), and all 21 patients were positive for IgG. B19V DNA was detected in blood by PCR in 20 patients (95%). Nerve biopsy showed necrotizing small‐vessel vasculitis in one patient (17%), perivascular lymphocytic and macrophagic infiltrates in five (83%), and B19V DNA was detected by PCR in all four tested nerves. Most patients received immunomodulatory treatment (*n* = 19; 90%). MM relapse occurred in four patients (19%). A partial recovery was observed in 17/20 patients (85%), two remained stable (10%), and one achieved complete recovery (5%).

**Conclusions:**

B19V infection should be systematically investigated in patients presenting with MM, especially in young individuals (including children) with predominantly sensory symptoms, predominant upper limb nerve involvement, and/or cranial nerve involvement.

## Graphical abstract

Parvovirus B19 infection can cause multiple mononeuropathy in young and immunocompetent patients, predominantly affecting sensory nerves, with frequent involvement of the median, fibular, and ulnar nerves, as well as occasional cranial nerve impairment. Viral DNA was detected in blood and nerve tissue, and nerve biopsy findings supported an inflammatory, sometimes vasculitic, mechanism. Most patients improved following immunomodulatory therapy, although relapses occurred in a minority.

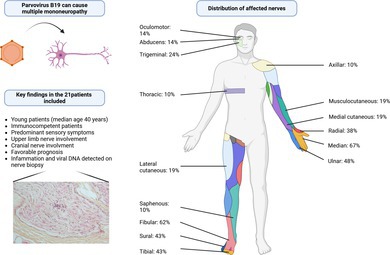

## 1 Introduction

Parvovirus B19 (B19V) is a small DNA virus responsible for infections that are frequently asymptomatic [1]. However, it has been associated with a wide range of clinical manifestations, including erythema infectiosum (fifth disease), transient aplastic crises in children with sickle cell anemia, chronic pure red cell aplasia, nonimmune hydrops fetalis, arthritis, and myocarditis [1, 2].

Neurological manifestations related to B19V infection have also been reported. Central nervous system involvement includes meningitis, encephalitis, cerebellar ataxia, and transverse myelitis [1, 2, 3]. Peripheral nervous system manifestations are less common but include Guillain‐Barré syndrome, carpal tunnel syndrome, and neuralgic amyotrophy [1, 2, 3]. Even more rarely, there have been some reports of clinical and electrophysiological features consistent with multiple mononeuropathy (MM; also named mononeuropathy multiplex), a syndrome characterized by the asymmetric, non‐length‐dependent involvement of at least two peripheral nerves [4, 5, 6, 7, 8].

To date, fewer than 10 cases of MM associated with B19V infection have been reported [4, 5, 6, 7, 8]. These cases suggest a predominantly sensory presentation with subacute onset and relatively favorable outcomes, although some patients experienced persistent sensory deficits.

Given the rarity of these reports, data on the clinical characteristics and prognosis of MM secondary to B19V infection remain limited. In the present study, we therefore aimed to better characterize the clinical, electrophysiological, and prognostic features of this syndrome by describing a multicenter cohort of patients with B19V‐related MM.

## 2 Methods

### 2.1 Study Design and Participants

In this retrospective, observational, multicenter study, 20 university neuromuscular reference centers in France and one in Switzerland were contacted to identify those with B19V‐related MM patients diagnosed between January 2015 and January 2025. Seven centers (Lyon, Nancy, Paris—Pitié‐Salpêtrière, Paris—Kremlin‐Bicêtre, Lille, Marseille, Toulouse, and Lausanne) reported cases and participated in the study. Inclusion criteria were: (1) a diagnosis of MM confirmed by electrodiagnostic (EDX) studies, including nerve conduction studies (NCS) and needle electromyography (EMG); and (2) evidence of B19V infection, defined by the presence of B19V IgM antibodies and/or detection of viral DNA in peripheral blood by polymerase chain reaction (PCR). The diagnosis of MM was defined as the presence of asymmetric, non–length‐dependent axonal involvement of multiple individual nerves, with focal reductions in compound muscle action potentials (CMAP) and/or sensory nerve action potentials (SNAP) amplitudes affecting distinct nerve territories, possibly associated with multifocal active denervation at rest and/or a neurogenic pattern during voluntary contraction on needle EMG [9]. Patients with only B19V IgG antibodies were excluded. All patients included in the present study underwent B19V testing as part of the MM diagnostic workup. All clinical data were collected anonymously from the patients' medical records. Patients were provided with an information letter, in accordance with French law; Swiss patients signed an informed consent allowing data reuse. All procedures involving human participants were conducted in compliance with the ethical standards of the Hospices Civils de Lyon (HCL ethics approval #25‐5010) and the 1964 Declaration of Helsinki and its later amendments.

### 2.2 Clinical, Laboratory and Electrophysiological Data

Demographic data collected were sex, immune status (immunocompetent or immunocompromised), age at symptom onset, and duration of follow‐up. Clinical variables of interest were extra‐neurological manifestations, type of initial neurological symptoms, mode of symptom onset (acute vs. subacute/chronic), sensory symptoms (paresthesia, hypoesthesia), motor weakness, neuropathic pain, and cranial nerve involvement. Acute onset was defined as a symptom nadir occurring within four weeks, and subacute/chronic onset as a nadir occurring after four weeks. The overall Neuropathy Limitations Scale (ONLS) score, the modified Rankin Scale (mRS) score, and the need for walking assistance were collected at both the initial and final visits. Disease course was retrospectively assessed by the authors, based on the medical records, and categorized as stable, partial recovery, or complete recovery. Data on immunomodulatory treatments and use of pain medication were collected. Results from EDX studies and laboratory investigations—blood tests (C‐reactive protein [CRP], B19V IgM, IgG, and DNA), and cerebrospinal fluid (CSF) analysis (white blood cell count, protein level, presence of oligoclonal bands, and B19V DNA)—were also collected. Blood B19V DNA levels were categorized as high (≥ 6 log IU/mL), moderate (≥ 4 to < 6 log IU/mL), or low (< 4 log IU/mL) viral load [10, 11, 12]. On NCS, sensory nerve involvement was defined as a sensory nerve action potential amplitude below the laboratory's lower limit of normal, and motor nerve involvement as a compound muscle action potential amplitude below the laboratory's lower limit of normal. Nerve involvement was defined clinically for cranial and thoracic nerves, and based on both clinical and electrophysiological data for limb nerves. Results from muscle, nerve and skin biopsies, including detection of B19V DNA by PCR and relevant histopathological findings, were collected. B19V DNA levels in nerve tissue were considered significant above 6 log IU/mL.

### 2.3 Statistical Analyses

Statistical analyses were performed using Excel (v16.93.1, Microsoft, Redmond, WA, US) and Prism (v10.4.2, GraphPad software, San Diego, CA, US). Categorical variables were described as counts and percentages, and quantitative variables were described using medians and interquartile ranges [IQR]. Comparisons between patients were done using the Mann–Whitney test for continuous variables and Fisher's exact test for categorical variables.

## 3 Results

### 3.1 Epidemiological and Clinical Characteristics

A total of 21 patients were included. Two patients had been previously published as case reports [7, 8]. Twelve were female (57%), and all were immunocompetent. The median age at symptom onset was 40 years [IQR: 31–44] (Table 1). Nineteen (90%) patients experienced initial symptoms before 60 years of age, including three (14%) before 18 years of age (two at 14 years of age in two patients, and one at 16 years of age). In these three pediatric cases, the diagnosis was established after referral to an adult neuromuscular specialist. Twelve patients (57%) had extra‐neurological manifestations (Table 1), occurring prior to neurological symptoms in six (29%), a median 1 week [IQR: 1–4] before, and simultaneously in the other six (29%). Cutaneous manifestations included purpuric lesions of the lower limbs in five patients, erythematous papular lesions of the trunk in one, and generalized pruriginous erythema in one. All patients initially presented with sensory symptoms, and four (19%) reported neuropathic pain at onset. Most patients (*n* = 13; 62%) presented with a subacute/chronic disease onset (Table 1). Moreover, the symptom onset was sequential in 19 (90%) and monophasic in two (10%). During neurological events, all patients reported sensory symptoms, 19 (90%) reported neuropathic pain, nine (43%) developed motor weakness, and seven (33%) had cranial nerve involvement (Table 1). Symptoms were asymmetric in 20 patients (95%). One patient developed autonomic dysfunction, presenting with tachycardia as well as erectile and urinary dysfunction.

**TABLE 1:** Epidemiological and clinical characteristics of patients with multiple mononeuropathy secondary to parvovirus B19 infection.

	Total population (*n* = 21)
Female, *n*/*N* (%)	12/21 (57)
Immunocompetent patients (%)	21/21 (100)
Age at first symptoms, median [IQR]	40 [31–44]
Extra‐neurological manifestations^a^, *n*/*N* (%)	12/21 (57)
Weight loss	7/21 (33)
Rash	7/21 (33)
Asthenia	5/21 (24)
Fever	2/21 (10)
Arthralgia	2/21 (10)
Anemia	1/21 (5)
First neurological symptoms, *n*/*N* (%)	
Sensory symptoms	21/21 (100)
Neuropathic pain	4/21 (19)
Symptoms onset, *n*/*N* (%)	
< 4 weeks	8/21 (38)
≥ 4 weeks	13/21 (62)
Symptoms during neurological events, *n* *N* (%)	
Sensory symptoms	21/21 (100)
Neuropathic pain	19/21 (90)
Motor weakness	9/21 (43)
Cranial nerve involvement	7/21 (33)

^a^ A patient could have several extra‐neurological manifestations.

**FIGURE 1:**
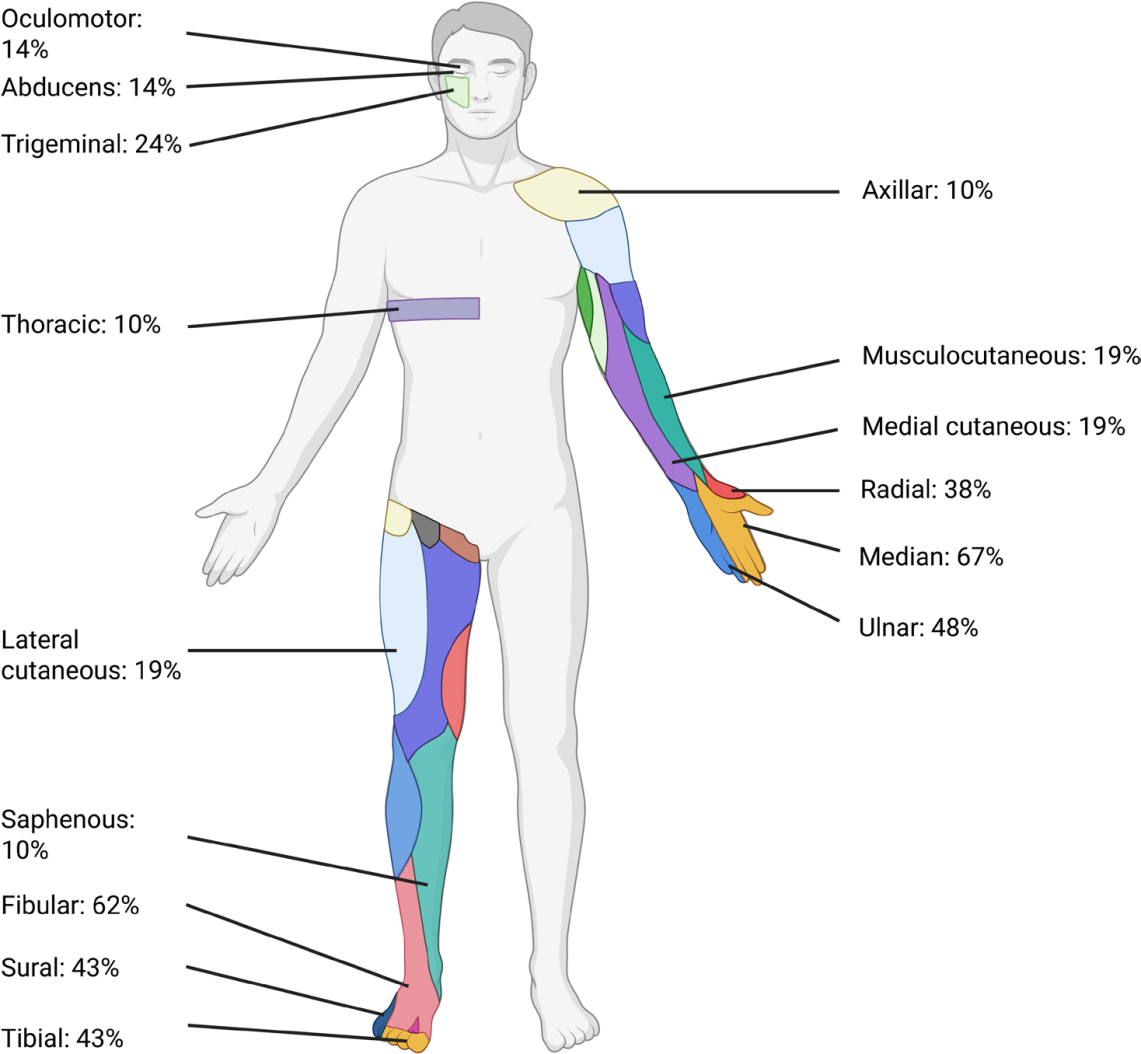
Distribution of affected nerves in patients with multiple mononeuropathy secondary to parvovirus B19 infection. The percentage of patients in whom each specific nerve was affected is indicated (created in BioRender, Theuriet, J. 2025, https://BioRender.com/yeabykx).

In the 21 patients, the most frequently involved nerves were the median (*n* = 14; 67%), fibular (*n* = 13; 62%), ulnar (*n* = 10; 48%), sural (*n* = 9; 43%), tibial (*n* = 9; 43%), and radial (*n* = 8; 38%) nerves. Cranial nerves were involved in 7 patients (33%): the trigeminal (*n* = 5; 24%), oculomotor (*n* = 3; 14%), and abducens (*n* = 3; 14%) nerves (Table S1 and Figure 1). Limb nerves were affected in both upper and lower limbs in 13 patients (62%), only in upper limbs in four patients (19%), and only in lower limbs in four patients (19%). Of the 109 limb nerves involved, 61 (56%) were in the upper limbs and 48 (44%) in the lower limbs.

### 3.2 Paraclinical Characteristics

On NCS, all patients had sensory nerve involvement, which was isolated in seven cases (33%); 14 patients (67%) had both sensory and motor involvement (Table 2). NCS findings in major nerves are detailed in Table S2. All electrophysiological patterns were axonal, with no evidence of demyelination in any patient. The median number of affected sensory and/or motor nerves per patient was 4 [IQR: 3–7] (Table 2). Three patients (14%) had at least one motor nerve that was unexcitable, and 10 patients (48%) had at least one sensory nerve that was unexcitable. Needle EMG revealed abnormal spontaneous activity in 12 patients (57%) and a neurogenic recruitment pattern in 13 (62%; Table 2).

**TABLE 2:** Paraclinical characteristics of patients with multiple mononeuropathy secondary to parvovirus B19 infection.

	Total population (*n* = 21)
Nerve conduction study	
Sensory nerve involvement, *n*/*N* (%)	21/21 (100)
Pure sensory nerve involvement, *n*/*N* (%)	7/21 (33)
Sensory and motor nerves involvement, *n*/*N* (%)	14/21 (67)
Number of involved nerves, median [IQR]	4 [3–7]
Needle EMG, *n*/*N* (%)	
PSWs and/or fibrillation potentials at rest	12/21 (57)
Neurogenic pattern during contraction^a^	13/21 (62)
Blood analyses	
Time from symptom onset in weeks, median [IQR]	7 [4–12]
Positive B19V IgM, *n*/*N* (%)	12/20 (60)
Positive B19V IgG, *n*/*N* (%)	20/20 (100)
Positive B19V PCR, *n*/*N* (%)	20/21 (95)
Viral load ≥ 4 log IU/mL, *n*/*N* (%)	6/18 (33)
Elevated CRP, *n*/*N* (%)	1/21 (5)
CSF analyses, *n*/*N* (%)	
Pleocytosis (white cell count ≥ 5 cells/μL)	4/19 (21)
Elevated proteins (> 0.5 g/L)	6/19 (32)
Oligoclonal bands (≥ 2)	1/15 (7)
Positive B19V PCR	3/12 (25)
Skin biopsy, *n*/*N* (%)	
Small‐vessel vasculitis with vessel wall fibrinoid necrosis	3/5 (60)
Positive B19V PCR	1/2 (50)
Normal (%)	2/5 (40)
Nerve biopsy, *n*/*N* (%)	
Small‐vessel vasculitis with vessel wall fibrinoid necrosis	1/6 (17)
Lymphocytic and macrophagic perivascular infiltrates	5/6 (83)
Positive B19V PCR	4/4 (100)

Abbreviations: B19V, parvovirus B19; CRP, C‐reactive protein; EMG, electromyography; PCR, polymerase chain reaction; PSW, positive sharp waves.

^a^ Characterized by reduced recruitment with or without long‐duration large motor unit action potentials.

No alternative cause of MM or vasculitis was identified in any of the patients; antineutrophil cytoplasmic antibodies (ANCA) were negative in all patients, and antinuclear antibodies (ANA) were negative in 16 patients (76%). In the five patients with positive ANA, no disease‐specific autoantibodies were detected. One patient (5%) had elevated CRP (without skin lesions). B19V IgM antibodies were present in 12/20 patients (60%), and all 21 patients were positive for IgG. In blood, B19V DNA was detected by PCR in 20 patients (95%; Table 2). Viral loads were < 6 log IU/mL in all patients, ranging from 2.4 to 5.5 log IU/mL. A moderate viral load (≥ 4 log IU/mL) was less frequently observed (6/18 patients, 33%) than low viral load (< 4 log IU/mL; 12/18 patients, 67%; Table 2). The patient with a negative blood PCR had a positive PCR in the muscle biopsy. These blood tests were performed after a median 7 weeks from symptom onset [IQR: 4–12]. The median interval from symptom onset to blood test was longer in patients without IgM antibodies (median 8 weeks; [IQR: 6–28]) compared to the patients with positive IgM antibodies (median 6 weeks; [IQR: 2–8]), but the difference was not significant (*p* = 0.12).

CSF analysis showed pleocytosis in 4/19 patients (21%), ranging from 5 to 19 cells/mm^3^, elevated protein levels in 6/19 (32%), ranging from 0.62 to 1.08 g/L, oligoclonal bands in 1/15 (7%), and B19V DNA detected by PCR in 3/12 (25%; Table 2).

**FIGURE 2:**
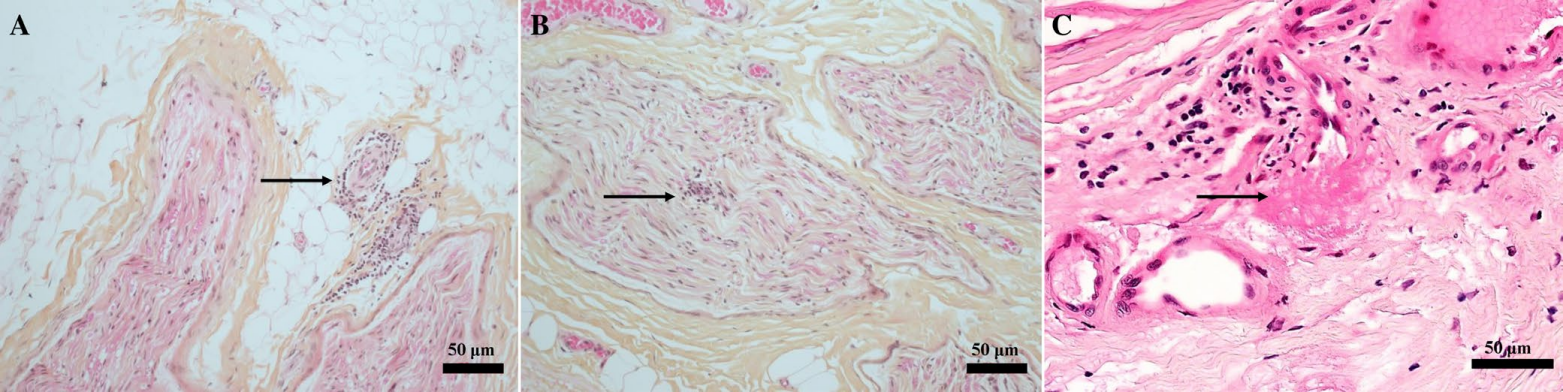
Histological findings from three nerve biopsies. (A) Superficial fibular nerve biopsy showing epineurial perivascular lymphocytic and macrophagic infiltrates (arrow; formalin fixed tissue, hematoxylin–eosin‐saffron; HES stained, 200×). (B) Sural nerve biopsy showing endoneurial infiltrates (arrow; formalin fixed tissue, HES stained, 200×). (C) Superficial fibular nerve biopsy showing fibrinoid necrosis of a small epineurial artery wall (arrow; formalin fixed tissue, hematoxylin–eosin; HE stained, 400×).

Skin biopsies performed on purpuric lesions in three patients revealed necrotizing small‐vessel vasculitis, whereas the two biopsies performed on clinically normal skin to search for vasculitic lesions showed normal findings. A nerve biopsy was performed in six patients. A necrotizing small‐vessel vasculitis was observed in one patient (17%), and perivascular lymphocytic and macrophagic infiltrates in the other five (83%; Table 2; Figure 2).

B19V DNA was detected by PCR in nerve in 4/4 patients (Table 2), including 2/4 patients with no B19V IgM. In 3/4 patients, the viral load in the nerve was significant (> 6 log IU/mL), while it was low in the blood (< 4 log IU/mL). Additionally, 2/2 patients (100%) had B19V DNA detected by PCR in the muscle, and 1/3 in the skin (33%).

### 3.3 Treatment and Prognosis

Most patients received immunomodulatory treatment (*n* = 19; 90%), and most received neuropathic pain medication (*n* = 14; 67%; Table 3). In 11 patients (52%), intravenous immunoglobulins (IVIg) were combined with oral corticosteroids (CS) during the disease course. Among them, two also received intravenous CS. IVIg and oral corticosteroids were combined from the start in 9/11 patients, while two patients were initially treated with intravenous CS alone and subsequently received IVIg due to relapse. IVIg was used alone in five patients (24%), while oral CS were used alone in three patients (14%) during the disease course, one of whom also received intravenous CS. No other immunomodulatory treatment was used. The median number of IVIg courses was three [IQR: 1–6]. The dose was 2 g/kg per course in all but one patient, who received 1 g/kg. The median duration of oral CS treatment was 6 months [IQR: 3–11]. The initial dose was 1 mg/kg/day in 11/14 patients (79%), 0.5 mg/kg/day in 2/14 patients (14%), and 1.5 mg/kg/day in the last patient (7%). Among the three patients who received intravenous CS, one received a course of three 1 g infusions, another received a course of five 1 g infusions, and the third received a course of three 500 mg infusions. No immunosuppressive treatment other than corticosteroids was used.

**TABLE 3:** Treatment and prognosis in patients with multiple mononeuropathy secondary to parvovirus B19 infection.

	Total population (*n* = 21)
Scores at first evaluation	
ONLS: total, median [IQR]	2 [2–4]
Upper limbs, median [IQR]	2 [1–2]
Lower limbs, median [IQR]	1 [0–3]
Walking aid, *n*/*N* (%)	1/21 (5)
mRS, median [IQR]	1 [1–2]
Immunomodulatory treatment, *n*/*N* (%)^a^	19/21 (90)
IVIg	16/21 (76)
Oral corticosteroids	14/21 (67)
Intravenous corticosteroids	3/21 (14)
Neuropathic pain medications, *n*/*N* (%)	14/21 (67)
Number of patients with relapse, *n*/*N* (%)	4/21 (19)
Time to last follow‐up in months, median [IQR]	12 [6–17]
Disease course, *n*/*N* (%)	
Stable	2/20 (10)
Partial recovery	17/20 (85)
Complete recovery	1/20 (5)
Symptoms at last visit, *n*/*N* (%)	
Sensory symptoms	17/20 (85)
Motor weakness	7/20 (35)
Pain	7/20 (35)
Cranial nerve involvement	2/20 (10)
Scores at last visit	
ONLS: total, median [IQR]	2 [1–3]
Upper limbs, median [IQR]	1 [1–2]
Lower limbs, median [IQR]	0 [0–1]
Walking aid, *n*/*N* (%)	0/20 (0)
mRS, median [IQR]	1 [1–2]

Abbreviations: IVIg, intravenous immunoglobulin; mRS, modified Rankin scale; ONLS, overall neuropathy limitations scale.

^a^ Patients could receive combined treatment.

At first evaluation, the median ONLS score was 2 [IQR: 2–4], the median mRS score was 1 [IQR: 1–2], and one patient (5%) required a walking aid (Table 3). Nine patients (43%) had an mRS score ≥ 2. At last follow‐up (median 12 months [IQR: 6–17]), the median ONLS score was 2 [IQR: 1–3], and the median mRS score remained at 1 [IQR: 1–2]. Five patients (25%) had an mRS score ≥ 2, and no patient required a walking aid (Table 3). A partial recovery was observed in 17/20 patients (85%), while two remained stable (10%) and one achieved complete recovery (5%; Table 3). The two patients who did not receive immunomodulatory treatments reported either stability or a partial recovery, and treated patients reported stability (1/18, 6%), partial (16/18, 89%), or complete (1/18, 6%) recovery.

MM relapse occurred in four patients (19%). They occurred in 3/5 patients (60%) initially treated with CS alone, and in 1/14 patients (7%) initially treated with IVIg ± CS (*p* = 0.04). No relapse occurred in the two untreated patients. Two patients had a single relapse, and two had two relapses. The six relapses occurred after a median 11 months [IQR: 6–15] after disease onset (Table 3). Five (83%) involved a single new nerve, while one (17%) involved two new nerves. Five relapses (83%) occurred during corticosteroid (CS) treatment, and one (17%) occurred a few days after discontinuation of CS. The median oral CS dose at the time of relapse was 7 mg [IQR: 2–26]. Three relapses (50%) occurred without prior IVIg treatment, while three relapses occurred following IVIg infusions (2 and 6 months after a single infusion, and 2 months after a ninth course). In the three relapses with available PCR results, the B19V viral load had slightly decreased or remained stable at the time of relapse compared to the initial measurement: 2.73 vs. 2.00 log IU/mL, 4.63 vs. 4.44 log IU/mL, 3.98 vs. 3.61 log IU/mL, at an interval of, respectively, 12, 6, and 15 months.

## 4 Discussion

We describe herein the main clinical, paraclinical, therapeutic, and prognostic features of a cohort of 21 patients presenting with MM associated with parvovirus B19V infection, representing the largest cohort of B19V‐related MM reported to date.

Several clinical characteristics observed in these patients may help distinguish B19V‐related MM from other vasculitic neuropathies (VN), which remain the most common cause of MM [13]. First, the median age at symptom onset in the cohort presented herein was approximately 40 years, which is lower than the ~60 years typically reported in other VN [14, 15, 16]. Notably, only 10% of the patients of the present study were older than 60 years of age at disease onset, and three patients presented with symptoms before the age of 18 years. This contrasts with the rarity of reported pediatric MM cases [17, 18]. Pediatric B19V‐related MM has not yet been reported, possibly due to diagnostic challenges, as suggested by the need for referral to an adult neuromuscular specialist observed in the three pediatric cases herein. In our opinion, B19V‐related MM could be an underestimated cause of MM, particularly in the pediatric population. Second, all patients presented initially with sensory symptoms, and a third had a pure sensory neuropathy on EDX study, in line with previous reports of B19V‐associated MM [4, 5, 6]. In addition, the pattern of nerve involvement also appeared to differ from that typically seen in VN. The median nerve was particularly affected in the present study, while in other VN, the fibular and tibial nerves are more commonly involved [16, 19, 20]. Furthermore, upper limb nerves were more frequently affected than lower limb nerves in patients studied herein. In contrast, upper‐limb predominance is rare in VN—for example, it is observed in only 5% of non‐systemic VN [16]. Finally, some atypical sensory nerves that are classically spared in VN were affected in patients herein, such as the medial cutaneous nerve of the arm, the lateral cutaneous nerve of the thigh, or the saphenous nerve. Cranial nerve involvement, already reported in B19V‐related MM, also appears to be a distinctive feature [4, 5]; it was observed in a third of cases herein, which appears more frequent than in other VN such as non‐systemic form (around 5%), and granulomatosis with polyangiitis (around 15%) [16, 21]. Importantly, neither the absence of systemic symptoms of B19V infection, nor an immunocompetent status should lead clinicians to rule out this diagnosis, as previously suggested [2, 3].

All patients had positive IgG serology for B19V, which is consistent with the known persistence of IgG over time [22]. The absence of IgM in over a third of patients may weaken the causal inference between B19V and MM, but this could be explained by the delay between symptom onset and testing [23], and more importantly, two patients were IgM negative but PCR positive in the nerve. Moreover, no other etiology of MM was identified in the patients. It is of note that the detection of viral DNA by PCR in blood was positive in all but one patient, confirming its diagnostic value. However, previous studies have shown that B19V DNA at low levels (< 4 log IU/mL) can persist in blood long after the acute phase [3, 5]. Detection of B19V DNA in nerve tissues may be of greater diagnostic value, as PCR signals revealed significant DNA concentrations in nerve tissue while blood DNA levels showed only moderate or low viral load. Taken together, these blood and nerve PCR results suggest that, at the time of sampling, the B19V infection had started several weeks earlier, with ongoing direct viral involvement of the nerve tissue.

CRP was elevated in only one case in the present study, in contrast to other VN, such as polyarteritis nodosa, where CRP is elevated in nearly all cases, and other forms of VN, where elevation is observed in approximately a third to half of cases [14, 21]. In addition, CSF analysis was frequently normal, with few inflammatory signs or positive B19V PCR, which may reflect the predominantly distal distribution of affected nerves that are anatomically remote from nerve roots and CSF compartments.

The prognosis of B19V‐related MM appears favorable. At baseline, disability scores were modest, and they remained low at follow‐up. However, most patients continued to experience residual symptoms, particularly sensory disturbances, which can be disabling in this young population and can negatively affect quality of life. Relapses may occur but were observed in a minority of cases (around a fifth in the present study). Three patients who experienced relapse had not received IVIg before the first relapse and were treated with CS alone, and the fourth had only one course of IVIg in association with CS. Moreover, one patient from the present cohort, previously reported, experienced worsening with CS but improved with IVIg [7]. Thus, IVIg could represent an interesting first‐line therapy, as in other B19V complications such as red cell aplasia [24, 25], although the optimal treatment duration for B19V‐related MM remains unknown. Several considerations may temper this proposal. Most patients were treated, but the two untreated individuals remained stable or improved spontaneously. Moreover, the specific effect of IVIg or CS is difficult to evaluate because they were frequently administered together. However, both these treatments remain the cornerstone for the treatment of clinical complications of B19V infection, and CS is the principal treatment of VN [21, 26]. Larger studies are needed to better evaluate the effect of the different therapeutic options in B19V‐related MM.

The findings from nerve biopsies are interesting as they allow some insight into potential pathophysiological mechanisms. The combination of B19V DNA detection with high viral load, with histological evidence of tissue vasculitis or perivascular lymphocytic/macrophagic infiltration suggests an immune‐mediated response to the virus. This may result in nerve damage through direct involvement of vessel walls or via inflammation in adjacent structures.

The present study does, however, have certain limitations, in addition to the sample size discussed above. Because of the retrospective nature of the present study, paraclinical investigations were not standardized, and some data were missing. Moreover, the nerve biopsy procedure was not standardized across centers. However, variability in biopsy size is unlikely to significantly affect DNA load, as a one‐log difference would require a biopsy approximately ten times larger. Nonetheless, differences in buffer volume and elution steps can influence quantification, since variations in extraction protocols may introduce bias. Therefore, direct comparison of DNA loads between biopsies is not fully reliable, although values exceeding 6 log IU/mL can be considered meaningful.

In conclusion, B19V infection should be systematically investigated by serology and PCR testing in patients presenting with MM, even in the absence of systemic symptoms, especially in young individuals (including children) with predominantly sensory symptoms, cranial nerve involvement, and/or predominant upper limb nerve involvement.

## Author Contributions

**Julian Theuriet:** conceptualization, writing – original draft, methodology, writing – review and editing, formal analysis, data curation, project administration, investigation, visualization. **Maud Michaud:** writing – review and editing, data curation. **Guillaume Fargeot:** writing – review and editing, data curation. **Céline Labeyrie:** writing – review and editing, data curation. **Anaïs Grosset:** writing – review and editing, data curation. **Maude Bucy:** writing – review and editing, data curation. **Ludivine Kouton:** writing – review and editing, data curation. **Florian Hubben:** writing – review and editing, data curation. **Véronique Manel:** writing – review and editing, data curation. **Florent Cluse:** writing – review and editing, data curation. **Adrien Bohic:** writing – review and editing, data curation. **Nicolas Rodriguez:** writing – review and editing, data curation. **Philippe Petiot:** writing – review and editing, data curation. **Geneviève Billaud:** writing – review and editing, data curation. **Vincent Fabry:** writing – review and editing, data curation. **Pascal Cintas:** writing – review and editing, data curation. **Thierry Maisonobe:** writing – review and editing, data curation. **Karine Viala:** writing – review and editing, data curation. **Rabab Debs:** writing – review and editing, data curation. **Dimitri Psimaras:** writing – review and editing, data curation. **Sarah Leonard‐Louis:** writing – review and editing, data curation. **Benjamin Terrier:** writing – review and editing, data curation. **Alina Dorobat:** writing – review and editing, data curation. **Céline Tard:** writing – review and editing, data curation. **Stéphane Darteyre:** writing – review and editing, data curation. **Alex Vicino:** writing – review and editing, data curation. **Marie Théaudin:** writing – review and editing, data curation. **Clovis Adam:** writing – review and editing, data curation, visualization. **Françoise Bouhour:** writing – review and editing, data curation. **Timothée Lenglet:** writing – review and editing, data curation. **Grégory Destras:** writing – review and editing, data curation. **Nathalie Streichenberger:** writing – review and editing, data curation, visualization. **Antoine Pegat:** conceptualization, investigation, writing – review and editing, methodology, supervision, data curation.

## Funding

The authors have nothing to report.

## Conflicts of Interest

Centre Hospitalier Universitaire Vaudois (CHUV) received for M.T. travel grants and fees for advisory boards from Takeda and CSL Behring. A.P. has received consultancy fees, congress travel support, and meeting participation fees from CSL Behring. J.T. has received consultancy fees from CSL Behring. C.T. reports personal fees and nonfinancial support from LFB (Laboratoire Français du Biomédicament), and nonfinancial support from CSL Behring. F.C. reports travel congress travel support and meeting participation fees from CSL Behring and LFB. P.C. has received honoraria for lectures from CSL Behring and LFB. G.F. reports meeting participation fees from CSL Behring and LFB. F.B. reports meeting participation fees from CSL Behring. K.V. reports consultancy fees from CSL Behring and meeting participation fees from LFB. R.D. reports consultancy fees from CSL Behring and meeting participation fees from LFB. T.L. reports personal fees and travel support from LFB.

## Supporting information


**Appendix S1:** ene70565‐sup‐0001‐Tables.pdf.

## Data Availability

De‐identified data may be made available to the corresponding author upon reasonable request.
