# Supplementary material for: Multiple Mononeuropathy Secondary to Parvovirus B19 Infection: A Case Series
Source: Eur J Neurol. 2026 Mar 20;33(3):e70565. doi: 10.1111/ene.70565 (PMC13093382; doi:10.1111/ene.70565)
Supplement: Supplementary file 1 — Appendix S1: ene70565‐sup‐0001‐Tables.pdf. [file ENE-33-e70565-s001.pdf]

Supplementary Table 1: Nerves affected in patients with multiple mononeuropathy secondary to parvovirus B19 infection.

Nerve, n/N (%)	Unilateral	Bilateral	Total population (n=21)
Median	4	10	14/21 (67%)
Fibular	8	5	13/21 (62%)
Ulnar	5	5	10/21 (48%)
Sural	6	3	9/21 (43%)
Tibial	6	3	9/21 (43%)
Radial	6	2	8/21 (38%)
Trigeminal	5	0	5/21 (24%)
Medial cutaneous nerve of the arm	3	1	4/21 (19%)
Musculocutaneous	4	0	4/21 (19%)
Lateral cutaneous nerve of the thigh	4	0	4/21 (19%)
Oculomotor	2	1	3/21 (14%)
Abducens	3	0	3/21 (14%)
Axillar	1	1	2/21 (10%)
Saphenous	2	0	2/21 (10%)
Thoracic	2	0	2/21 (10%)

Nerve involvement was defined clinically for cranial and thoracic nerves and based on both clinical and electrophysiological data for limb nerves.

Supplementary Table 2: Nerve conduction study findings.

**Patient** (age at exam, years)	**Side**	**Motor nerve conduction**								**Sensory nerve conduction**							
		Median nerve		Ulnar nerve		Common fibular nerve		Tibial nerve		Median nerve		Ulnar nerve		Superficial fibular nerve		Sural nerve	
		Amp (mV) (>4)	CV (m/s) (>45)	Amp (mV) (>4)	CV (m/s) (>45)	Amp (mV) (>2)	CV (m/s) (>40)	Amp (mV) (>4)	CV (m/s) (>40)	Amp (µV), (>18)	CV (m/s) (>45)	Amp (µV) (>18)	CV (m/s) (>45)	Amp (µV) (>8)	CV (m/s) (>40)	Amp (µV) (>10)	CV (m/s) (>40)
1 (43)	**R**	7.5	58.1	NA	NA	8.9	49.3	5.6	NA	34.2	58.4	29.7	58.1	17.7	51.5	29.7	65.1
	**L**	5.8	50.2	NA	NA	10	50.4	4.6	NA	26.1	58.1	24.5	60.6	10.8	48.8	31.2	50.3
2 (40)	**R**	11.6	45.2	12.1	49.8	11.2	**38.9**	7.1	NA	**11.6**	54.6	NA	NA	8.6	**33.5**	**5.4**	**31.6**
	**L**	11.1	55.4	10.5	46.0	6.6	44.0	10.7	NA	**9.6**	52.0	NA	NA	**7.0**	**30.6**	**7.3**	**35.2**
3 (41)	**R**	9.5	52.1	8.3	66.1	9.6	48.3	12.9	NA	**3.9**	57.6	NA	NA	**7.1**	**37.7**	28.5	**36.6**
	**L**	9.5	55.5	7.0	63.5	6.5	43.8	5.3	NA	**11.8**	48.8	NA	NA	8.8	40.8	18.5	**38.1**
4 (42)	**R**	**NR**	**NR**	NA	NA	NA	NA	NA	NA	**NR**	NA	NA	NA	NA	NA	NA	NA
	**L**	NA	NA	**NR**	**NR**	**NR**	**NR**	**NR**	**NR**	NA	NA	NA	NA	**NR**	NA	NA	NA
5 (30)	**R**	9.6	55.4	15.1	63.1	2.1	47.2	17.7	NA	**8.1**	50.2	NA	NA	19.7	40.9	10.1	43.4
	**L**	11.4	57.3	15.6	61.4	3.9	49.6	16.6	NA	19.8	54.5	NA	NA	**NR**	**NR**	16.8	40.4
6 (55)	**R**	**2.8**	53.9	5.8	55.3	3.3	44.3	9.5	50.1	**4.0**	62.0	**6.9**	62.2	NA	NA	**2.9**	44.0
	**L**	**2.5**	53.3	7.7	55.1	**NR**	**NR**	5.1	48.6	**5.1**	63.5	**6.7**	61.7	NA	NA	**8.2**	50.2
7 (15)	**R**	10.5	49	11.1	63.0	3.2	46.0	NA	NA	40.6	54.9	57.2	**61.1**	**NR**	**NR**	13.4	47.0
	**L**	NA	NA	NA	NA	3.2	NA	11.1	43.0	NA	NA	NA	NA	22.9	46.2	22.6	42.5
8 (39)	**R**	13.6	56.4	13.0	56.0	**NR**	**NR**	6.9	**39.4**	23.0	45.8	**5.9**	51.2	**NR**	**NR**	**4.4**	41.3
	**L**	4.6	56.2	10.3	53.1	**1.4**	**39.4**	**NR**	**NR**	18.3	47.7	**5.3**	52.1	**NR**	**NR**	**NR**	**NR**
9 (39)	**R**	4.3	50.2	5.9	51.8	**1.2**	**31.4**	6.5	NA	**9.3**	58.0	**6.2**	50.9	NA	NA	13.9	47.5
	**L**	6.6	46.2	7.9	57.6	2.3	**38.0**	4.2	**38.3**	**8.0**	58.8	18.0	67.1	9.1	43.6	14.5	**36.3**
10 (16)	**R**	15.2	54.7	13.4	56.7	11.1	44.3	21.1	NA	**9.4**	58.0	26.7	68.2	9.4	NA	37.6	NA
	**L**	7.9	48.2	9.8	56.1	3.0	**37.0**	12.6	NA	36.6	57.7	**11.4**	60.0	21.4	NA	57.0	46.3
11 (44)	**R**	5.4	55.3	5.9	65.8	7.3	49.6	14.3	53.5	**11.0**	54.0	30.5	57.3	**2.4**	47.5	17.8	50.5
	**L**	**2.1**	NA	7.0	NA	6.3	49.6	7.4	58.7	28.3	49.9	26.1	56.3	**7.8**	44.8	19.2	49.6

**Patient** (age at exam, years)	**Side**	**Motor nerve conduction**								**Sensory nerve conduction**							
		Median nerve		Ulnar nerve		Common fibular nerve		Tibial nerve		Median nerve		Ulnar nerve		Superficial fibular nerve		Sural nerve	
		Amp (mV) (>4)	CV (m/s) (>45)	Amp (mV) (>4)	CV (m/s) (>45)	Amp (mV) (>2)	CV (m/s) (>40)	Amp (mV) (>4)	CV (m/s) (>40)	Amp (µV), (>18)	CV (m/s) (>45)	Amp (µV) (>18)	CV (m/s) (>45)	Amp (µV) (>8)	CV (m/s) (>40)	Amp (µV) (>10)	CV (m/s) (>40)
12 (47)	**R**	7.5	58.9	7.8	59.0	4.6	44.6	10.8	49.5	62.0	55.0	39.0	48.0	13.0	45.0	10.0	50.0
	**L**	8.0	58.2	7.9	55.0	3.1	46.4	13.6	44.7	70.0	54.0	22.0	45.0	8.0	44.0	**5.0**	41.0
13 (41)	**R**	**0.6**	51.1	11.4	60.3	4.8	44.6	11.0	45.5	**NR**	**NR**	42.0	60.0	12.0	52.0	10.0	49.0
	**L**	8.4	55.6	8.8	57.0	5.0	44.2	8.3	43.5	33.0	45.0	34.0	52.0	15.0	52.0	10.0	42.0
14 (14)	**R**	8.8	49.6	7.2	52.1	6.3	47.2	11.0	47.2	33.0	65.0	31.0	48.0	13.0	46.0	14.0	42.0
	**L**	12.7	55.4	8.0	63.1	3.3	41.7	6.1	41.7	55.0	51.0	40.0	48.0	**4.0**	**35.0**	**8.0**	38.0
15 (32)	**R**	5.7	49.4	6.4	62.6	6.8	40.9	NA	NA	25.0	49.0	49.0	53.0	31.0	58.0	NA	NA
	**L**	**2.6**	NA	13.3	54.2	8.5	46.7	12.5	51.0	33.0	58.0	71.0	52.0	24.0	43.0	15.0	47.0
16 (37)	**R**	13.9	55.4	8.2	60.4	NA	NA	NA	NA	83.0	54.0	94.0	54.0	NA	NA	NA	NA
	**L**	**0.6**	52.8	7.5	64.6	NA	NA	NA	NA	**2.0**	46.0	84.0	51.0	NA	NA	NA	NA
17 (40)	**R**	12.3	55.0	10.4	60.0	**1.6**	**35**	13.6	NA	72.0	46.0	19.4	NA	45.8	**37.0**	36.7	**38.0**
	**L**	8.7	56.0	11.3	63.0	**1.6**	61.0	15.4	NA	64.0	47.0	**15.0**	NA	34.6	**38.0**	29.9	41.0
18 (27)	**R**	8.6	58.0	9.7	59.0	5.8	63.0	22.2	NA	74.0	51.0	45.9	61.0	36.2	44.0	23.8	51.0
	**L**	7.5	56.0	10.6	56.0	4.9	44.0	17.9	NA	59.1	63.0	41.6	65.0	**6.2**	**38.0**	**NR**	**NR**
19 (37)	**R**	NA	NA	NA	NA	3.2	46.0	12.2	50.0	NA	NA	NA	NA	25.6	NA	**8.8**	43.0
	**L**	NA	NA	NA	NA	7.2	51.0	13.9	NA	NA	NA	NA	NA	38.5	45.0	28.2	43.0
20 (72)	**R**	6.6	53.4	5.6	51.6	7.1	52.2	5.9	48.7	**NR**	**NR**	**NR**	**NR**	14.9	43.9	17.5	52.1
	**L**	7.2	54.5	7.2	62.3	4.8	41.3	11.2	45.7	20.7	60.6	**3.2**	44.6	13.3	49.5	17.6	50.3
21 (62)	**R**	**0.7**	62.3	NA	NA	NA	NA	NA	NA	**5.1**	**34.0**	NA	NA	NA	NA	NA	NA
	**L**	**1.3**	48.0	NA	NA	NA	NA	NA	NA	**NR**	**NR**	NA	NA	NA	NA	NA	NA

Amp: amplitude, CV: conduction velocity, R: right, L: left, NA: not available, NR: no response. In bold: abnormal result according to normal laboratory values.
